# Influenza vaccine effectiveness against influenza A in children based on the results of various rapid influenza tests in the 2018/19 season

**DOI:** 10.1371/journal.pone.0249005

**Published:** 2021-03-26

**Authors:** Masayoshi Shinjoh, Norio Sugaya, Yoshio Yamaguchi, Ichiro Ookawara, Yuji Nakata, Atsushi Narabayashi, Munehiro Furuichi, Naoko Yoshida, Akinobu Kamei, Yuu Kuramochi, Akimichi Shibata, Motoko Shimoyamada, Hisataka Nakazaki, Naohiko Maejima, Erika Yuasa, Eriko Araki, Naonori Maeda, Takuma Ohnishi, Mitsuhiro Nishida, Nobuhiko Taguchi, Makoto Yoshida, Kenichiro Tsunematsu, Meiwa Shibata, Yasuhiro Hirano, Shinichiro Sekiguchi, Chiharu Kawakami, Keiko Mitamura, Takao Takahashi

**Affiliations:** 1 Department of Pediatrics, Keio University School of Medicine, Tokyo, Japan; 2 Department of Pediatrics, Keiyu Hospital, Kanagawa, Japan; 3 Institute of Clinical Research & Department of Infection and Allergy, National Hospital Organization Tochigi Hospital, Tochigi, Japan; 4 Department of Pediatrics, Japanese Red Cross Shizuoka Hospital, Shizuoka, Japan; 5 Department of Pediatrics, Nippon Koukan Hospital, Kanagawa, Japan; 6 Department of Pediatrics, Kawasaki Municipal Hospital, Kanagawa, Japan; 7 Department Pediatrics, Yokohama Municipal Citizen’s Hospital, Kanagawa, Japan; 8 Department of Pediatrics, Subaru Health Insurance Society Ota Memorial Hospital, Gunma, Japan; 9 Department of Pediatrics, Japanese Red Cross Ashikaga Hospital, Tochigi, Japan; 10 Department of Pediatrics, Saitama City Hospital, Saitama, Japan; 11 Department of Pediatrics, Tokyo Dental College Ichikawa General Hospital, Chiba, Japan; 12 Department of Pediatrics, Tokyo Metropolitan Ohtsuka Hospital, Tokyo, Japan; 13 Department of Pediatrics, Saiseikai Utsunomiya Hospital, Tochigi, Japan; 14 Department of Pediatrics, National Hospital Organization Tokyo Medical Center, Tokyo, Japan; 15 Department of Pediatrics, National Hospital Organization Saitama Hospital, Saitama, Japan; 16 Department of Pediatrics, Shizuoka City Shimizu Hospital, Shizuoka, Japan; 17 Department of Pediatrics, Sano Kosei General Hospital, Tochigi, Japan; 18 Department of Pediatrics, Hino Municipal Hospital, Tokyo, Japan; 19 Department of Pediatrics, Yokohama Rosai Hospital, Kanagawa, Japan; 20 Department of Pediatrics, Hiratsuka City Hospital, Kanagawa, Japan; 21 Yokohama City Institute of Public Health, Yokohama, Japan; 22 Department of Pediatrics, Eiju General Hospital, Tokyo, Japan; The Chinese University of Hong Kong, HONG KONG

## Abstract

During influenza epidemics, Japanese clinicians routinely conduct rapid influenza diagnostic tests (RIDTs) in patients with influenza-like illness, and patients with positive test results are treated with anti-influenza drugs within 48 h after the onset of illness. We assessed the vaccine effectiveness (VE) of inactivated influenza vaccine (IIV) in children (6 months–15 years old, N = 4243), using a test-negative case-control design based on the results of RIDTs in the 2018/19 season. The VE against influenza A(H1N1)pdm and A(H3N2) was analyzed separately using an RIDT kit specifically for detecting A(H1N1)pdm09. The adjusted VE against combined influenza A (H1N1pdm and H3N2) and against A(H1N1)pdm09 was 39% (95% confidence interval [CI], 30%–46%) and 74% (95% CI, 39%–89%), respectively. By contrast, the VE against non-A(H1N1)pdm09 influenza A (presumed to be H3N2) was very low at 7%. The adjusted VE for preventing hospitalization was 56% (95% CI, 16%–77%) against influenza A. The VE against A(H1N1)pdm09 was consistently high in our studies. By contrast, the VE against A(H3N2) was low not only in adults but also in children in the 2018/19 season.

## Introduction

In Japan, rapid influenza diagnostic tests (RIDTs) are considered core tools for determining whether or not to start treatment with anti-influenza drugs. During influenza epidemics, Japanese clinicians routinely use RIDTs in the examination of patients with influenza-like illness, and most patients with positive test results are treated with anti-influenza drugs within 48 h after the onset of illness [1, 2]. We have reported the vaccine effectiveness (VE) in children using a test-negative case-control (TNCC) design based on the results of RIDT since the 2013/14 season. Capitalizing on the advantage of the widespread use of RIDTs, we have been able to enroll over 3000 to 4000 children in our VE study every year [3–7]. Although the quality of RIDT tests was reported to be insufficient [8], they are clearly on the agenda of public health authorities, clinicians, and health services managers in Japan, and the sensitivity and specificities of the tests that we used were satisfactory [9, 10].

The purpose of this study was to measure the VE in the 2018/19 season based on the results of RIDTs, including the VE for preventing hospitalization and by vaccine dose. In addition, the VE against influenza A(H1N1)pdm09 was measured using an RIDT kit specifically for detecting A(H1N1)pdm09.

## Methods

To assess VE, we used a TNCC design based on RIDT results [3–7, 11]. Children who were 6 months to 15 years old who had a fever of ≥38°C and were suspected of having influenza and had received an RIDT at one of the outpatient clinics of 21 hospitals in Gunma, Tochigi, Saitama, Tokyo, Chiba, Kanagawa, and Shizuoka Prefectures between November 1, 2018 and March 31, 2019 were enrolled. All hospitals were located within a 70-mile radius of Tokyo. As cough and other respiratory tract–related symptoms are less prominent, especially in younger children, and as myalgias and malaise are not always seen in children [12], we did not include symptoms other than fever. Consecutive sampling or whole sampling for each collaborator was encouraged and performed, especially in hospitals with small sample size.

### Quadrivalent influenza vaccine strains used in the 2018/19 season

The vaccine strains in the 2018/19 season were A/Singapore/GP1908/2015 (A(H1N1)pdm09) (A/Michigan/45/2015-like), A/Singapore/INFIMH-16-0019/2016 (A(H3N2)), B/Phuket/3073/2013 (Yamagata lineage), and B/Maryland/15/2016 (Victoria lineage).

### The diagnosis of influenza

Nasopharyngeal swabs were obtained from patients. The swab for each test kit was inserted deeply into the back of at least one nostril, left in place for several seconds, rotated to absorb secretions, placed into the medium of each kit, and tested immediately. RIDT kits capable of differentiating between influenza A and influenza B were used in all hospitals, including the ImmunoAce FLU kit (TAUNS Laboratories, Inc., Shizuoka, Japan), Quick Chaser Flu A, B kit (Mizuho Medy Co., Ltd., Saga, Japan), QuickNavi-Flu kit (Denka Seiken Co., Ltd., Tokyo, Japan), Espline Influenza A&B-N kit (Fujirebio Inc., Tokyo, Japan), and Spotchem FLORA FluAB (Arkray Factory, Inc., Shiga, Japan). All of these kits have high sensitivity (approximately 85%–95%) and specificity (up to 100%) compared to reverse transcription polymerase chain reaction (RT-PCR) [6].

The ImunoAce Flu test is designed to not only detect influenza A or B, but also to detect A(H1N1)pdm09 with the use of an additional test kit (Linjudge FluA/pdm, TAUNS Laboratories, Inc., Shizuoka, Japan). Its sensitivity and specificity for A(H1N1)pdm09 were 97.6% (95% confidence interval [CI]: 87.4%–99.9%) and 92.6% (95%CI: 82.1%–97.9%) in adults, respectively [10]. Thus, by consecutively testing patients with the ImunoAce Flu test followed by the Linjudge FluA/pdm test, we were able to diagnose whether a patient had A(H1N1)pdm09 or A(H3N2) infection within a short time. In this study, we defined “A(H3N2)” as patients positive for influenza A but negative for A(H1N1)pdm09 in hospitals where the test kit Linjudge FluA/pdm had been used. However, it must be recognized that influenza A-positive with A(H1N1)pdm09-negative does not always mean A(H3N2)-positive.

### Case and control patient identification and VE

RIDT-positive patients were enrolled as cases and RIDT-negative patients were enrolled as controls. The information shown in Table 1 was collected and recorded. The source of vaccination information, including doses, was medical interviews and/or medical records from the Maternal and Child Health Handbooks provided by local governments [13]. As the handbooks are not digitalized, parents usually bring them to health visits. Patients who had already taken anti-influenza viral drugs prior to the visit were excluded. VE was defined as “1- odds ratio (OR),” and OR was calculated as follows:

**Table 1:** Characteristics of children enrolled in 2018/19 season (n = 4243)*.

Characteristics		Influenza A Positive (%) N = 2135	Influenza Negative (%) N = 2098	Difference between influenza A and influenza-negative p-value**
**Sex**	Female	971 (45)	915 (44)	*p* = 0.211
	Male	1162 (54)	1183 (56)	
	Not reported	2 (0)	0 (0)	
**Age**	6–11 months	66 (3)	140 (7)	*p* < 0.001
	1–2 years	339 (16)	697 (33)	
	3–5 years	530 (25)	560 (27)	
	6–12 years	980 (46)	571 (27)	
	13–15 years	220 (10)	130 (6)	
Comorbidity***	No	1811 (86)	1714 (83)	*p* = 0.005
	Yes	293 (14)	352 (17)	
**Month of onset**	November	11 (1)	142 (7)	*p* < 0.001
	December	235 (11)	454 (22)	
	January	1436 (67)	793 (38)	
	February	415 (19)	532 (25)	
	March	38 (2)	177 (8)	
Clinic visit*** **(hours after onset)**	<12 h	661 (33)	596 (33)	*p* < 0.001
	12–48 h	1259 (63)	1030 (57)	
	>48 h	67 (3)	178 (10)	
**Received vaccine during the season**	No	1368 (64)	1061 (51)	*p* < 0.001
	Yes	767 (36)	1037 (49)	
Vaccine doses during the season***	None	1368 (64)	1061 (51)	*p* < 0.001
	One	222 (10)	251 (12)	
	Two	535 (25)	775 (37)	
Treatment with antivirals***	No	61 (3)	1632 (97)	*p* < 0.001
	Any	1994 (97)	44 (3)	
Hospitalized after diagnosis at outpatient clinics***	**Unvaccinated**	48	59	
	**Vaccinated**	26	71	
Non-hospitalized after diagnosis at outpatient clinics***	**Unvaccinated**	1320	964	
	**Vaccinated**	740	896	

*: 10 children with influenza B were included in the 4243 enrolled patients.

**: Chi-square test

***: Some data were missing

(number of influenza-positives among vaccinated patients × number of influenza-negatives among unvaccinated patients) / (number of influenza-negatives among vaccinated patients × number of influenza-positives among unvaccinated patients).

Adjustments to the VE are explained below. The VE for preventing influenza A(H1N1)pdm09 was also analyzed in three hospitals where the ImunoAce Flu and the Linjudge FluA/pdm were used.

### VE for preventing hospitalization

In the TNCC design, the cases were patients who were hospitalized because of influenza (RIDT-positive hospitalized patients) after diagnosis at an outpatient clinic. Patients hospitalized because of influenza-like illness who were RIDT-negative served as the control group. The VE for preventing influenza hospitalization was estimated based on the vaccine coverage rate of both hospitalized patient groups.

### VE by vaccine dose (6 months–12 years old)

In Japan, a single dose is recommended for children ≥13 years old, but a two-dose regimen is recommended for all children aged 6 months to 12 years old [14]. Doses of 0.25 ml and 0.5 ml are recommended for children 6 months to 2 years old and ≥3 years old, respectively. Approximately 70% of vaccinated children 6 months to 12 years old receive two doses [15]. We recorded the number of vaccine doses per patient (none, one, or two) and compared the VE among them. The VE was calculated among all three groups (“none,” “one,” and “two”) using three combinations (1. one vs. none, 2. two vs. none, and 3. two vs. one).

### Statistical analyses and ethics

Statistical analyses were performed using the SPSS 25.0 software program (IBM, Chicago, USA) and the Ekuseru-Toukei 2015 for Windows software program (Social Survey Research Information Co., Ltd., Tokyo, Japan). *p* < 0.05 was considered statistically significant for all analyses.

To analyze the VE for both preventing influenza and preventing hospitalization, binary logistic regression methods were chosen. Confounding factors, such as age (0–15 years old), comorbidity (yes or no), colder or warmer area (northern, middle, or southern area of the Kanto region), and month of onset, were entered in the analysis by the forced entry method. For some analyses, the time tested after onset (<12, 12–48, and >48 h) was also entered. These factors for adjustment have been the same since our 2013/14 study [3]. The chi-square test was used to compare the test-positive and test-negative groups. To reduce the chance of type 1 errors, *p*-values were also Bonferroni corrected for the dose analysis (*p*-value <0.05/3 = 0.017 was also calculated in one table).

This study was approved by the Keio University Ethics Committee (Approval Number 20130216, revised in 2018). Eligible patients and their guardians were informed about the study objectives and methods verbally or via posters in outpatient clinics. We recorded the necessary information using a standardized questionnaire sheet. The requirement for obtaining written consent was waived by the Keio University Institutional Review Board because testing patients with an RIDT is a standard practice, and all questions regarding this study are essential in daily practice.

## Results

### The 2018/19 season in Japan

**Fig 1:**
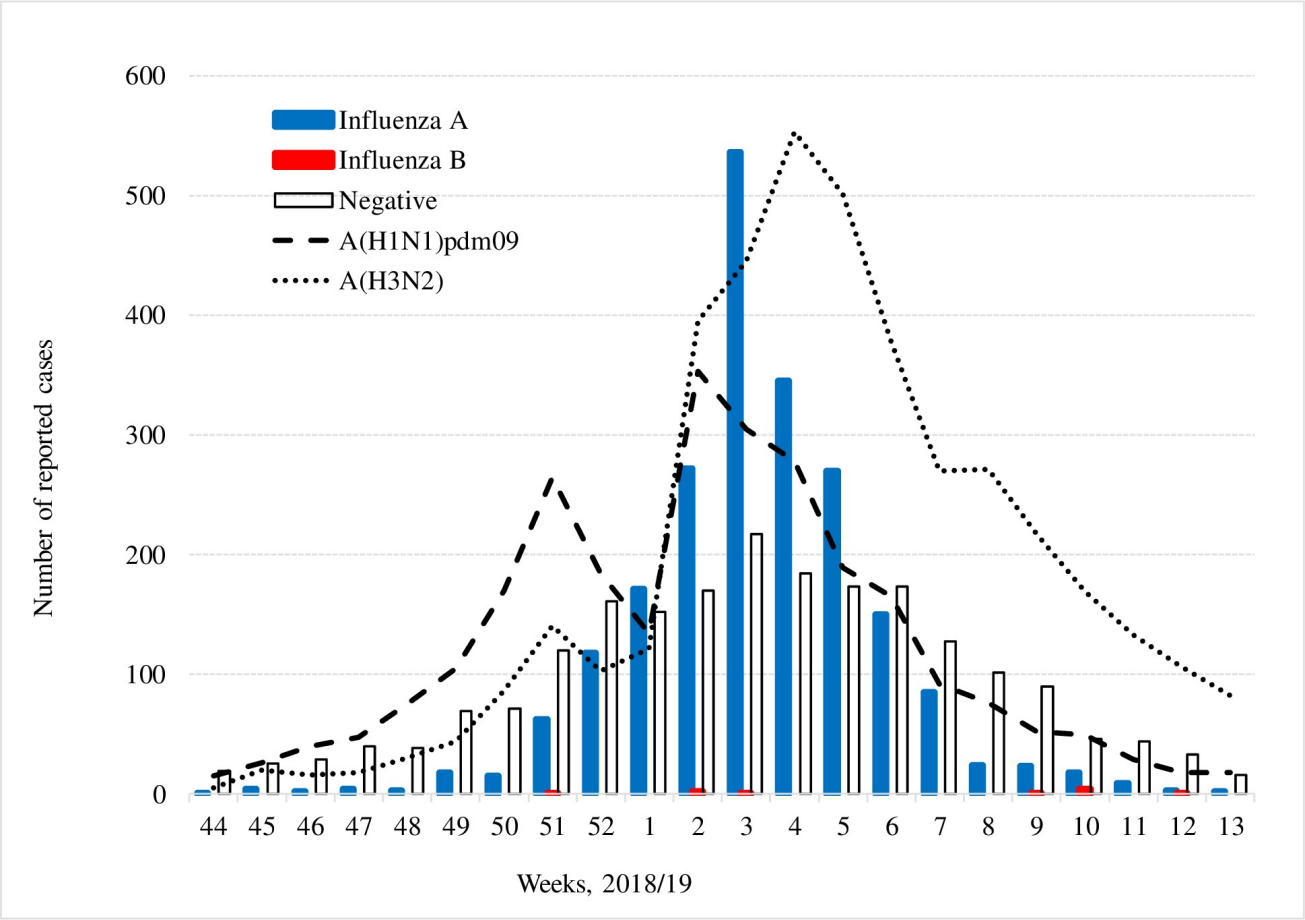
Number of patients tested by rapid influenza diagnostic tests by week in the column chart (n = 4243). Epidemic curves for A(H1N1)pdm09 and A(H3N2) by the World Health Organization in the line graph [16].

The change in the number of RIDT-positive or RIDT-negative patients is shown in Fig 1. The influenza epidemic in the 2018/19 season was a mixed epidemic of influenza A subtypes. A monophasic epidemic pattern of influenza A with a single peak in January (week 3 of 2020) was observed. According to the World Health Organization (WHO)’s website [16], the influenza viruses isolated/detected in the 2018/19 season consisted of A(H1N1)pdm09 (36%), A(H3N2) (56%), and type B (8%). Based on the Japanese sentinel surveillance report, the estimated number of influenza patients, including adults and children, who visited medical facilities between week 36 of 2018 and week 17 of 2019 was approximately 12 million, meaning that the scale of the epidemic was rather large this season [17]. All A(H1N1)pdm09 strains isolated were classified as 6B.1A, belonging to the same subclade of the vaccine strain A/Singapore/GP1908/2015 (A(H1N1)pdm09), and most of the A(H3N2) strains isolated were classified as 3C.2a, also belonging to the same subclade of the vaccine strain, A/Singapore/INFIMH-16-0019/2016 (A(H3N2)) [17].

### Characteristics of the enrollees in the 2018/19 season

A total of 4243 children 6 months to 15 years old were enrolled in the 2018/19 season (Table 1). Children who were hospitalized and children who were not hospitalized after diagnosis at outpatient clinics were included in these analyses. Among the 4243 enrollees who were eligible for inclusion in the analysis in this study, 2145 were RIDT-positive, including 2135 with influenza A and 10 with influenza B, and 2098 were RIDT-negative. As only 10 cases with influenza B were reported, VE against influenza B was not analyzed in this study.

Of the children with influenza A, 97% (1920/1987) had received an RIDT within 48 h of onset. Ninety-seven percent (1994/2055) of the children with influenza A were treated with antivirals (neuraminidase inhibitors or baloxavir) (Table 1). The overall vaccine coverage rates were 36% (767/2135), 30% (3/10), and 49% (1037/2098) for influenza A-positive, B-positive, and control patients (RIDT-negative), respectively (Table 1).

### VE for preventing influenza illness

The adjusted VE for preventing influenza A illness was 39% (95% CI, 30%–46%, N = 4233) (Table 2). The adjusted VE was 63% at 6 to 11 months (95% CI, 15%–84%, N = 206) and 56% at 1 to 2 years old (95% CI, 42%–67%, N = 1036) compared with 25% at 6 to 12 years old (95% CI, 6%–39%, N = 1551) and -5% at 13 to 15 years old (not significant; 95% CI, -74%–37%, N = 350).

**Table 2:** Adjusted vaccine effectiveness (VE) of quadrivalent inactivated influenza vaccine for preventing influenza in the 2018/19 season (n = 4243). VE for preventing influenza A (N = 4243).

Age group	VE% (95% CI)	Cases (vaccinated, unvaccinated)	Cases vaccinated, %	Controls [vaccinated, unvaccinated]	Controls vaccinated, %
**6 months–15 years**	39 (30–46) ^a^^,^ ^b^	(767, 1368)	36	[1037, 1061]	49
	38 (29–46) ^a^^,^ ^b^^,^ ^c^				
**6–11 months**	63 (15–84) ^a^	(9, 57)	14	[38, 102]	27
**1–2 years**	56 (42–67) ^a^	(128, 211)	38	[401, 296]	58
**3–5 years**	49 (35–61) ^a^	(201, 329)	38	[303, 257]	54
**6–12 years**	25 (6–39) ^a^	(365, 615)	37	[255, 316]	45
**13–15 years**	-5 (-74–37) ^a^	(64, 156)	29	[40, 90]	31

a. Adjusted for comorbidity (yes or no), area (northern, middle, or southern area of the Kanto region), month of onset

b. Adjusted for age (0–15 years old)

c. Adjusted for time tested after onset (<12, 12–48, and >48 h)

Of the 238 cases in 3 hospitals, 36 (10 vaccinated and 26 unvaccinated) cases were A(H1N1)pdm09-positive, 71 (44 vaccinated and 27 unvaccinated) cases were A(H1N1)pdm09-negative (defined as A(H3N2)), and 131 cases were influenza-negative. The adjusted VE for preventing influenza A was 37% (95% CI, -11%–64%, N = 238) by ImmunoAce FLU, and the adjusted VE for preventing influenza A(H1N1)pdm09 was 74% (95% CI, 39%–89%, N = 167) by Linjudge FluA/pdm. The VE in preventing influenza A(H3N2) was 7% (95% CI, -77%–51%, N = 202) (Table 3).

**Table 3:** Adjusted vaccine effectiveness (VE) of quadrivalent inactivated influenza vaccine for preventing influenza in the 2018/19 season (n = 4243). Adjusted vaccine effectiveness (VE) of quadrivalent inactivated influenza vaccine for preventing influenza A(H1N1)pdm09 and A(H3N2) in 3 hospitals that used rapid influenza diagnostic test kits for detecting A(H1N1)pdm09 (N = 238).

Type of influenza	VE% (95% CI) ^a^	Cases (vaccinated, unvaccinated)	Cases vaccinated, %	Controls [vaccinated, unvaccinated]	Controls vaccinated, %
Influenza A	37 (-11–64)	(54, 53)	50	[84, 47]	64
A(H1N1)pdm09	74 (39–89)	(10, 26)	28	[84, 47]	64
A(H3N2)	7 (-77–51)	(44, 27)	62	[84, 47]	64

a. Adjusted for comorbidity (yes or no), area (northern, middle, or southern area of the Kanto region), month of onset, and age (0–15 y/o).

### VE for preventing hospitalization against influenza A

There were 205 hospitalized children (74, 1, and 130 in influenza A, B, and RIDT-negative, respectively), and 3929 were not hospitalized (2060, 9, and 1860 in influenza A, B, and RIDT-negative, respectively) after diagnosis at outpatient clinics (Table 1). The adjusted VE for preventing hospitalization was 56% (95% CI, 16%–77%) (Table 4). Among 204 hospitalized cases and controls, the vaccine coverage was 35% (26/74) and 55% (71/130), respectively (Table 4). Among the 74 hospitalized children with influenza A, 8 (11%) and 10 (13%) had underlying neurological diseases (such as epilepsy) and respiratory diseases (such as asthma), respectively, and 33 (67%, 33/49) had seizures on admission.

**Table 4:** Adjusted vaccine effectiveness (VE) of quadrivalent inactivated influenza vaccine for preventing influenza hospitalization in the 2018/19 season.

Type of influenza	VE% (95% CI) ^a^	Cases (vaccinated, unvaccinated)	Cases vaccinated, %	Controls [vaccinated, unvaccinated]	Controls vaccinated, %
Influenza A	56 (16–77)	(26, 48)	35	[71, 59]	55

a. Adjusted for comorbidity (yes or no), area (northern, middle, or southern area of the Kanto region), month of onset, and age (0–15 y/o).

### VE by vaccine dose (6 months–12 years old)

The number of enrollees among influenza-positive and influenza-negative children by age group is shown in Table 5. In general, both the one- and two-dose regimens significantly reduced cases with influenza A (Table 6, “one vs. none,” “two vs. none”). Only the two-dose regimen was effective for preventing influenza A in 6 to 11 month olds (adjusted VE, 62% [95% CI, 9%–84%]) (*p*-value <0.05) and in 3 to 5 year olds (adjusted VE, 53% [95% CI, 38%–64%]) (Bonferroni-corrected α *p*-value <0.05/3). There was no significant difference between the one- and two-dose regimens.

**Table 5:** Adjusted vaccine effectiveness (VE) of quadrivalent inactivated influenza vaccine by vaccine dose for preventing influenza illness among children in the 2018/19 season. Number of enrollees.

	Influenza A Positive, n (%)				Influenza Negative, n (%)			
vaccine doses	0	1	2	Total	0	1	2	Total
**6–11 months old**	57 (86)	2 (3)	7 (11)	66 (100)	102 (73)	5 (4)	33 (24)	140 (100)
**1–2 years old**	211 (63)	18 (5)	108 (32)	337 (100)	296 (43)	81 (12)	313 (45)	690 (100)
**3–5 years old**	329 (63)	46 (9)	151 (29)	526 (100)	257 (46)	52 (9)	248 (45)	557 (100)
**6–12 years old**	615 (63)	98 (10)	263 (27)	976 (100)	316 (55)	76 (13)	178 (31)	570 (100)
**total**	1212 (64)	164 (9)	529 (28)	1905 (100)	971 (50)	214 (11)	772 (39)	19570)

**Table 6:** Adjusted vaccine effectiveness (VE) of quadrivalent inactivated influenza vaccine by vaccine dose for preventing influenza illness among children in the 2018/19 season. Adjusted vaccine effectiveness (VE) against influenza A by vaccine dose.

Age group^a^	Vaccine doses	VE% (95% CI)
**6–11 months**	one vs. none	65 (-210–96)
	two vs. none	62 (9–84)*
	two vs. one	-24 (-1223–89)
**1–2 years old**	one vs. none	68 (43–82)**
	two vs. none	53 (37–65)**
	two vs. one	-57 (-179–12)
**3–5 years old**	one vs. none	34 (-2–58)
	two vs. none	53 (38–64)**
	two vs. one	27 (-15–54)
**6–12 years old**	one vs. none	32 (5–52)*
	two vs. none	23 (2–39)*
	two vs. one	-13 (-63–21)
**6 months–2 years old**	one vs. none	66 (42–80)**
**(for 0.25 ml/dose)**	two vs. none	51 (37–63)**
	two vs. one	-51 (-164–14)
**Total** ^**b**^	one vs. none	41 (26–53)**
	two vs. none	42 (32–50)**
	two vs. one	-9 (-40–15)

a. Adjusted for comorbidity (yes or no), area (northern, middle, or southern area of the Kanto region), month of onset

b. Adjusted for comorbidity (yes or no), area (northern, middle, or southern area of the Kanto region), month of onset, and age

*p-value <0.05

**p-value <0.05/3 = 0.017 (Bonferroni-corrected)

## Discussion

In the 2018/19 season, a relatively low VE against influenza A (i.e., 39%; 95% CI, 30%–46%, N = 4233) was shown in this study, when the ratio of isolated strains for A(H1N1)pdm09 and A(H3N2) was approximately 40% vs. 60% in Japan [17]. Although the VE against influenza A(H1N1)pdm09 was high (74%; 95% CI, 39%–89%, N = 167), the VE against influenza A(H3N2) was very low (7%; 95% CI, -77%–51%, N = 202). Therefore, the low VE against influenza A was probably due to a markedly reduced VE against influenza A(H3N2).

Although we previously reported low VEs against influenza A infection during influenza epidemics mostly caused by the A(H3N2) subtype, such as 37% in 2014/15 (99% of influenza A strains identified were A(H3N2)) [4] and 38% in 2016/17 (97% of influenza A strains identified were A(H3N2)) [6], the degree of reduction in the VE against influenza A was not as extensive in children. Based on our experience, we concluded that the VE of IIV against A(H3N2) was low but still effective in children. In contrast, the VE against A(H3N2) was very low or not significant in adults in recent seasons [18, 19]. We recognized for the first time in this study that the very low VE against A(H3N2) was a serious problem not only in adults but also in children in the 2018/19 season.

In the 2018/19 season, the antigenicity of most A(H3N2) strains isolated in Japan was similar to the vaccine strain A/Singapore/INFIMH-16-0019/2016 (A(H3N2)) (genetic clade 3C.2a1) [17]. However, mutations in the egg-adapted vaccine strain of the A/Singapore/INFIMH-16-0019/2016 (A(H3N2)) were suspected, which may lead to very low or no VE [17].

The VE against A(H1N1)pdm09 was consistently high in our studies. We previously reported high VEs against A(H1N1)pdm09, such as 77% in the 2013/14 season, using RIDT kits specifically for detecting A(H1N1)pdm09, and the VE increased with age: from 67% in the 1- to 2-year-old group, to 84% in the 3- to 5-year-old group, and 90% in the 6- to 12-year-old group [3]. A Clearline Influenza A/B/(H1N1)2009 kit (Alere Medical Co., Ltd., Tokyo, Japan) or the combination of ImunoAce Flu and Linjudge FluA/pdm was used to detect A(H1N1)pdm09 [3], but the former has since been withdrawn due to unstable results. According to the antigenic analysis of A(H1N1)pdm09 strains, the antigenicity of most isolated strains was similar to that of the vaccine strain A/Singapore/GP1908/2015 (A(H1N1)pdm09) (A/Michigan/45/2015-like) (genetic clade 6B.1) in the 2018/19 season [17].

Because we used RIDTs, digital immunoassays, or even rapid nucleic acid amplification tests, we were unable to diagnose influenza A subtypes separately, except when RT-PCR was performed. We believe that the combination of ImunoAce Flu and Linjudge FluA/pdm is highly useful for distinguishing between influenza A subtypes compared with RT-PCR, although the sensitivity and specificity of the combination of tests is lower than with RT-PCR [10]. This combination of tests obtains results much more quickly than RT-PCR, and numerous samples can be tested at the same time. The most important point is its cost, which, at approximately USD$10 per test, is much cheaper than RT-PCR [10].

Using RT-PCR, high VE against A(H1N1)pdm09 in children was reported worldwide in the 2018/19 season. In the US, where most of the isolated influenza type A strains were A(H1N1)pdm09, the VE against A(H1N1)pdm09 in children 6 months to 17 years old was 62% [20]. In Canada, the VE against A(H1N1)pdm09 in children 1 to 8 years old was as high as 91% [21]. In contrast, a low or non-significant VE against A(H3N2) in this season was also reported among adults and children [18, 19].

In the present study, the VE for children 6 to 11 months old was shown to be the highest among all age groups, although the VE in this age group has not been statistically significant in our previous studies. It is generally thought that, in children 6 to 11 months old, the VE may be lower than in older groups because the vaccine-induced antibody response is immature [22]. However, it was reported that the VE of two doses of trivalent IIV in subjects 6 months to <1 year old for the 2002/03 to 2007/08 seasons was the highest among all age groups [23]. More studies are needed to assess VE in infants 6 to 11 months old.

Although a meta-analysis showed no convincing evidence that the influenza vaccine reduces mortality, hospitalization rates, or serious complications in children [24], we reported that, in four of the five seasons from 2013 to 2018, the VEs for preventing hospitalization were significantly effective [3–5], and the five-season VEs for preventing hospitalization for any influenza, influenza A, and influenza B were 46% (95% CI, 33%–56%), 50% (95% CI, 36%–60%), and 31% (95% CI, 4%–51%), respectively [7]. Furthermore, in the present study, the adjusted VE for preventing hospitalization against influenza A was 56% (95% CI, 16%–77%, N = 205), regardless of the relatively low VE for preventing illness against influenza A (39%). Therefore, we presume that influenza vaccination is more important for preventing severe influenza than for preventing influenza illness altogether.

Another observation was that only a two-dose regimen was effective against influenza A in children 3 to 5 years old. In our combined analysis of five seasons (2013/14–2017/18), both one- and two-dose regimens were effective against influenza A in all seasons [7]. The low effectiveness of a single dose against influenza A in children 3 to 5 years old was likely caused by the very low VE against influenza A(H3N2), which was only 7% in this recent season. We believe that a two-dose regimen is appropriate in younger children, because the one-dose regimen has not always been found effective in our studies. This is consistent with previous findings [22, 25, 26].

The major limitation of our past studies has been that our diagnostic tools are RIDTs, not RT-PCR. The sensitivity of RIDTs was recently reported to be as low as 42.6% for influenza A and 33.2% for influenza B in adult patients, leading to a deep distrust of RIDTs in the world medical community [8], although there was an issue in that report, with the timing of the sample collection for the RIDTs was not mentioned. This is relevant, as the sensitivity of RIDTs is dependent on the viral load in the upper respiratory tract, and the viral titers of patients with influenza virus infection in the upper respiratory tract peak during the first couple of days after the onset of influenza infection before declining to undetectable levels within a week [27].

In contrast, in Japan, during the 2009 H1N1 pandemic, the sensitivity and specificity of the RIDT (Prolast Flu AB, Mitsubishi Chemical Medience Corporation, Tokyo, Japan) was determined to be 80.0% and 97.1% in adults and children, compared with RT-PCR [9]. In 2020, very high sensitivity, such as 97.1% of a widely used RIDT (ImunoAce Flu; TAUNS Laboratories, Inc., Shizuoka, Japan) was reported in adults [10]. Moreover, the WHO Agenda for Public Health noted that the reliability of rapid tests in Japan seems to be higher than that in other countries, as most patients are tested within 48 h of the onset of illness [28]. Although the imperfect specificity of RIDT contributes to bias in the test-negative design, VE studies using RIDT have been reported [29, 30].

In conclusion, during the mixed influenza A subtype epidemic in the 2018/19 season, the VE against influenza A was low (39%), but the VE against influenza A(H1N1)pdm09 was high (74%). By contrast, the VE against influenza A(H3N2) was very low (7%). This very low VE against A(H3N2) was a serious problem not only in adults but also in children and was attributed to mutations in the egg-adapted vaccine strain of A/Singapore/INFIMH-16-0019/2016 (A(H3N2)) as observed or reported previously [18, 19, 31, 32]. Non-egg-based influenza vaccines including cell-culture vaccines and those with nucleic acid technologies are promising [33].
